# The Effects of Endurance Exercise in Hypoxia on Acid-Base Balance, Potassium Kinetics, and Exogenous Glucose Oxidation

**DOI:** 10.3389/fphys.2019.00504

**Published:** 2019-05-16

**Authors:** Daichi Sumi, Nobukazu Kasai, Hiroto Ito, Kazushige Goto

**Affiliations:** ^1^Graduate School of Sports and Health Science, Ritsumeikan University, Kusatsu, Japan; ^2^Faculty of Sports and Health Science, Ritsumeikan University, Kusatsu, Japan

**Keywords:** hypoxia, endurance exercise, carbohydrate metabolism, acid-base balance, K^+^

## Abstract

**Purpose:**

To investigate the carbohydrate metabolism, acid–base balance, and potassium kinetics in response to exercise in moderate hypoxia among endurance athletes.

**Methods:**

Nine trained endurance athletes [maximal oxygen uptake (VO_2max_): 62.5 ± 1.2 mL/kg/min] completed two different trials on different days: either exercise in moderate hypoxia [fraction of inspired oxygen (FiO_2_) = 14.5%, HYPO] or exercise in normoxia (FiO_2_ = 20.9%, NOR). They performed a high-intensity interval-type endurance exercise consisting of 10 × 3 min runs at 90% of VO_2max_ with 60 s of running (active rest) at 50% of VO_2max_ between sets in hypoxia (HYPO) or normoxia (NOR). Venous blood samples were obtained before exercise and during the post-exercise. The subjects consumed ^13^C-labeled glucose immediately before exercise, and we collected expired gas samples during exercise to determine the ^13^C-excretion (calculated as ^13^CO_2_/^12^CO_2_).

**Results:**

The running velocities were significantly lower in HYPO (15.0 ± 0.2 km/h) than in NOR (16.4 ± 0.3 km/h, *P* < 0.0001). Despite the lower running velocity, we found a significantly greater exercise-induced blood lactate elevation in HYPO compared with in NOR (*P* = 0.002). The bicarbonate ion concentration (*P* = 0.002) and blood pH (*P* = 0.002) were significantly lower in HYPO than in NOR. There were no significant differences between the two trials regarding the exercise-induced blood potassium elevation (*P* = 0.87) or ^13^C-excretion (HYPO, 0.21 ± 0.02 mmol⋅39 min; NOR, 0.14 ± 0.03 mmol⋅39 min; *P* = 0.10).

**Conclusion:**

Endurance exercise in moderate hypoxia elicited a decline in blood pH. However, it did not augment the exercise-induced blood K^+^ elevation or exogenous glucose oxidation (^13^C-excretion) compared with the equivalent exercise in normoxia among endurance athletes. The findings suggest that endurance exercise in moderate hypoxia causes greater metabolic stress and similar exercise-induced elevation of blood K^+^ and exogenous glucose oxidation compared with the same exercise in normoxia, despite lower mechanical stress (i.e., lower running velocity).

## Introduction

Carbohydrate metabolism during endurance exercise in hypoxia has been evaluated using several parameters, including blood glucose, lactate response, and the respiratory exchange ratio (RER) (Friedmann et al., 2004; Katayama et al., 2010; Morishima et al., 2014). However, carbohydrate oxidation using the RER may be overestimated during exercise in hypoxia due to the lower oxygen uptake and hyperventilation (Ogawa et al., 2007; Ofner et al., 2014; Sharma et al., 2019). An evaluation of carbohydrate oxidation using a stable isotope (^13^C) is an alternative procedure to overcome this problem (Harvey et al., 2007; Blondin et al., 2010; Smith et al., 2010; Tremblay et al., 2010).

The energy supply via the glycolytic system is enhanced during endurance exercise in hypoxia (Buchheit et al., 2012; Sumi et al., 2018a). During exercise, lactate and hydrogen ions (H^+^) are produced in working muscle via the augmented energy supply from the glycolytic system and are subsequently released into the blood circulation by Na^+^/H^+^ exchanger isoform 1 and monocarboxylate transporters (Juel, 1997, 2006), which elicits metabolic acidosis (lower muscle pH). Furthermore, the augmented metabolic stress stimulates the opening of muscle ATP-sensitive K^+^ (K_ATP_) channels, which subsequently increases the K^+^ efflux from working muscle into the extracellular fluid (Davies, 1990; Nielsen et al., 2002). Therefore, endurance exercise in hypoxia with greater intramuscular and blood acidification may promote the accumulation of blood K^+^ compared with exercise in normoxia. In our previous study (Sumi et al., 2018b), we evaluated the effect of endurance exercise in hypoxia on acid-base balance and K+ responses among middle-long distance runners. However, in this study, we used cycling exercise although middle-long distance runners were recruited. Therefore, the subjects performed unaccustomed exercise modality compared with their daily exercise training (running), which may be associated with unexpected results from the hypothesis. The lower muscle pH and accumulation of K+ in extracellular fluid during exercise are considered factors limiting sustained power output during endurance exercise (Trivedi and Danforth, 1966; Sahlin et al., 1981; Fitts, 1994; Sejersted and Sjøgaard, 2000; Bangsbo and Juel, 2006; Allen et al., 2008), whereas these factors may augment training adaptations (Mohr et al., 2007; Iaia et al., 2008; Bangsbo et al., 2009). For instance, Mohr et al. (2007) compared the effects of two different intense training regimens on skeletal muscle ion transport proteins and exercise performance. Sprint endurance training (30 s runs), with greater disturbance of muscle ion homeostasis (higher blood lactate and K^+^ concentrations) during the training sessions, caused greater adaptation of Na^+^/H^+^ exchanger isoform 1 and the Na^+^/K^+^-ATPase α1 isoform. These adaptations also led to lower venous H^+^ and K^+^ concentrations during intensive running exercise and further improved exercise performance compared with a normal endurance training group. Because the training adaptations arise from repetition of the acute physiological responses in each training session, determination of the exercise-induced acid–base balance and K^+^ responses would greatly improve our understanding of the mechanism underlying the enhanced endurance exercise capacity after several weeks of endurance training in hypoxia (Dufour et al., 2006; Czuba et al., 2011, 2013, 2017).

Therefore, the present study evaluated the acid–base balance, K^+^ kinetics, and exogenous glucose oxidation in response to endurance (running) exercise in moderate hypoxia among endurance runners. We hypothesized that endurance exercise in moderate hypoxia would facilitate the exercise-induced decrease in blood pH, elevate the K^+^ concentration, and promote exogenous glucose oxidation compared with the same relative intensity of exercise in normoxia.

## Materials and Methods

### Subjects

Nine endurance athletes (middle-long distance runners who belonged to the same truck and filed club in university) participated in the study. Exclusion criteria were (1) an unhealthy person (all subjects were required to pass the medical check conducted in university every year), (2) no experience in hypoxic training at least 3 months before the present experiment. Their means and standard errors (SE) for age, height, and body mass were 20.7 ± 0.9 years, 172.5 ± 2.2 cm, and 61.6 ± 2.8 kg, respectively. All athletes were born and living at sea level, and they maintained specific middle-long distance running training 5 days per week (approximately 70 km per week). They gave written informed consent after being informed of the purpose and risks associated with the experiment. This study was approved by the Ethics Committee for Human Experiments at Ritsumeikan University, Japan.

### Experimental Design

The subjects visited the laboratory four times during the study. During the first and second visits, maximal oxygen uptake (VO_2max_) tests were completed using a treadmill (Valiant; Lode, Groningen, the Netherlands) in both normoxia [inspired oxygen fraction (FiO_2_) = 20.9%] and normobaric hypoxia (FiO_2_ = 14.5%, equivalent to a simulated altitude of 3000 m). Each test was separated by 3 days and randomized.

**FIGURE 1:**
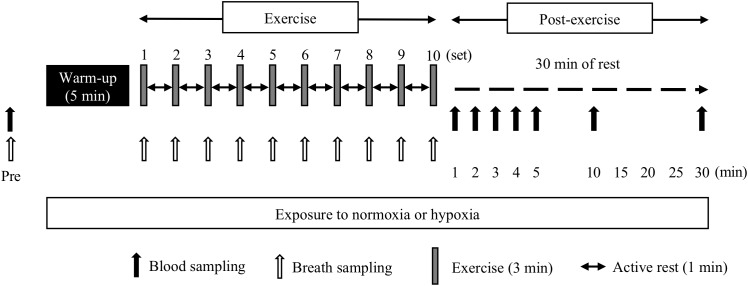
Overview of the study design.

During the third and fourth occasions, the subjects performed two experimental trials in either hypoxia (FiO_2_ = 14.5%, HYPO) or normoxia (FiO_2_ = 20.9%, NOR) on different days. We used a cross-over design and each trial was separated by 1 week. The two trials were started from the same time of the day, and the order of the two trials was randomized. As shown in Figure 1, all subjects completed high-intensity interval running on a treadmill at the same exercise intensity relative to VO_2max_ evaluated in hypoxia and normoxia. After completing the exercise, the subjects rested for 30 min under the respective condition (i.e., either normoxia or hypoxia). Changes in blood variables and the ^13^CO_2_/^12^CO_2_ ratio in the expired gas were monitored during and after the exercise to clarify the effects of exercise in hypoxia on the acid–base balance and potassium and exogenous glucose oxidation kinetics.

### Exercise Protocols

All exercise sessions in HYPO and NOR were conducted using a treadmill (Elevation series E95Ta; Life Fitness, Tokyo, Japan). Both trials were conducted in an environmentally controlled chamber. The hypoxic chamber used in this study was the whole-room type, and the hypoxic condition was established by nitrogen insufflation. The subjects ran for 5 min at 60% of VO_2max_ (warm-up exercise) from 15 min after entering the hypoxic chamber. From 5 min after the warm-up exercise, the subjects started an interval-type endurance exercise [10 × 3 min run at 90% of VO_2max_ separated by a 1 min run (active rest) at 50% of VO_2max_] in hypoxia or normoxia. This exercise regimen was designed to mimic the actual training regimen of well-trained endurance athletes (Niess et al., 2003; Sumi et al., 2018a,b). Each trial was separated by 1 week. The two trials started at the same time of the day, and the order of the two trials was randomized. To avoid psychological influences, the subjects were not informed about whether the trial was conducted in hypoxia or normoxia. Room temperature and humidity were set at 22°C and 50%, respectively.

### Maximal Oxygen Uptake in Normoxia or Hypoxia (Preliminary Measurement)

The initial running velocity was set at 12 km/h, and the running velocity was increased by 2 km/h every 2 min until it reached 16 km/h. Once the running velocity reached 16 km/h, it was increased by 0.6 km/h every minute until volitional exhaustion (Sumi et al., 2018a). During the test, expired gases were collected and analyzed using an automatic gas analyzer (AE300S; Minato Medical Science, Tokyo, Japan). The collected data were averaged every 30 s. Heart rate (HR) was measured continuously during the test using a wireless HR monitor (Accurex Plus; Polar Electro Oy, Kempele, Finland). The VO_2max_ tests were performed twice in either normoxia or hypoxia, and the order of the two VO_2max_ tests was randomized.

### Blood Variables

Following an overnight fast, the subjects visited the laboratory at 8:00 and rested before the first blood collection. A polyethylene catheter was inserted into an antecubital vein after a 20 min rest, and a baseline blood sample was obtained. Blood samples were collected 1, 2, 3, 4, 5, 10, and 30 min after the participants completed the exercise. All blood samples for determinations of blood gases and electrolytes were collected using a 2.5 mL syringe containing heparin. A 10 mL syringe was used to obtain serum and plasma samples. Serum and plasma samples were obtained after 10 min of centrifugation at 4°C (3000 rpm), and the samples were stored at −80°C until analysis.

The blood glucose, lactate, serum insulin, and ketone body concentrations as well as the blood-gas variables of the blood samples were measured. The blood-gas parameters, including the hydrogen ion concentration (pH), oxygen partial pressure (pO_2_), carbon dioxide partial pressure (pCO_2_), bicarbonate ion (HCO_3_^−^), and base excess (BE); the potassium (K^+^), sodium (Na^+^), and hemoglobin (Hb) concentrations; and the hematocrit (Hct) levels were measured using an automatic blood-gas analyzer (OPTI CCA TS, Sysmex, Hyogo, Japan). The exercise-induced plasma volume shift (%) was calculated using the Dill and Costill (1974) equation as follows:

$$\Delta \mathrm{PV}(\%)=100\times [({\mathrm{Hb}}_{\mathrm{pre}}/{\mathrm{Hb}}_{\mathrm{post}})\times (100-{\mathrm{Hct}}_{\mathrm{post}})/(100-{\mathrm{Hct}}_{\mathrm{pre}})-1],$$

where Hct is in % and Hb is in g/dL.

These analyses were completed within 15 min after blood collection, and the samples were put on ice until analysis. The blood glucose and lactate concentrations were measured using a glucose analyzer (Free style; Nipro, Osaka, Japan) and a lactate analyzer (Lactate Pro; ARKRAY, Kyoto, Japan) immediately after blood collection. The serum insulin and ketone body concentrations were measured at a clinical laboratory (SRL, Tokyo, Japan). The intra-assay coefficients of variability were 3.2 and 2.9% for the serum insulin and ketone body concentrations, respectively.

### Exogenous Glucose Oxidation Kinetics

Immediately before beginning the exercise, the subjects consumed 500 mg of ^13^C-glucose (D-Glucose-U-^13^C6, ^13^C: 99 atom%; Chlorella Industry, Tokyo, Japan) dissolved in 100 mL of purified water. In the ^13^C-glucose, the carbon atoms at all six positions in each glucose molecule were labeled with ^13^C. Before consuming the ^13^C-glucose, a baseline breath sample was collected using a 1.3 L sampling bag (Otsuka Pharmaceutical, Tokyo, Japan). Ten breath samples were collected until the end of the exercise in each set. The ^13^CO_2_/^12^CO_2_ ratio in the sample bag was evaluated using an infrared spectrometer (POC one; Otsuka Pharmaceutical). The ^13^CO_2_/^12^CO_2_ ratio was expressed as the absolute increase between samplings during exercise and at baseline.

The measured ^13^CO_2_ and ^12^CO_2_ abundance ratio was converted into the actual amount of excreted ^13^C and then converted using the formula to evaluate the ^13^C kinetics. The ^13^C excretion per unit time was calculated using the following equation (Schneider et al., 1978; Tanaka et al., 2013):

$${}^{13}C-\text{excretion}=(\Delta {\%}^{13}\text{C}/100)\times 300\times \text{BSA}.$$

Body surface area (BSA) can be estimated using the formula proposed by Du Bois and Du Bois (1916):

$$\text{BSA}=(W^{0.425}\times H^{0.725})\times 0.007184,$$

where *W* is the body weight measured in kilograms, and *H* is the body height measured in centimeters.

### Cardiorespiratory Variables and RPE

The oxygen uptake (VO_2_), carbon dioxide output (VCO_2_), RER, and expired minute ventilation (VE) were determined breath by breath in repetitions (reps) 5 and 10 of the interval exercise, and the average values of the respiratory variables during the final 1 min of each rep were calculated. Percutaneous oxygen saturation (SpO_2_) was measured at reps 5 and 10 during the interval exercise using a finger pulse oximeter (Smart Pulse; Fukuda Denshi, Tokyo, Japan) placed on the tip of the right forefinger. HR was recorded every 5 s during exercise; the average values were calculated during the final 1 min of each 3 min rep. The subjects indicated their rating of perceived exertion for respiratory strain (RPE-R) and leg muscle strain (RPE-L) at the end of each exercise rep using a 10-point scale to measure perceived exertion (Wilson and Jones, 1991).

### Energy Expenditure and Substrate Oxidation

The energy expenditure (EE) during exercise was calculated using the Weir (1949) equation, where VO_2_ and VCO_2_ are expressed as L/min. The values of VO_2_ and VCO_2_ were the averages during the final 1 min in rep 10 during the interval exercise:

$$\mathrm{Energy expenditure}\;(\text{kcal}/\text{min})=3.9\times {\text{VO}}_{2}+1.1\times {\text{VCO}}_{2}.$$

The rates of carbohydrate and fat oxidation were calculated using the following equations (Peronnet and Massicote, 1991; Manetta et al., 2002), where VO_2_ and VCO_2_ are expressed as L/min. VO_2_ and VCO_2_ values were the averages of the last 1 min of rep 10 during the interval exercise:

$$\text{Carbohydrate }(\text{g}/\text{min})=4.585\times {\text{VCO}}_{2}-3.226\times {\text{VO}}_{2}$$

$$\text{Fat }(\text{g}/\text{min})=1.695\times {\text{VO}}_{2}-1.701\times {\text{VCO}}_{2}.$$

### Statistical Analyses

Data are expressed as means ± SE. Two-way analysis of variance (ANOVA) with repeated measures was used to test the interaction (trial × time) and main effects (trial, time). When the ANOVA revealed a significant interaction or main effect, the Tukey–Kramer test was performed as a *post hoc* analysis to identify differences. The area under the curve (AUC) for ^13^C-excretion was compared between the two trials using a paired *t*-test. For all tests, *P* < 0.05 were considered to indicate statistical significance.

## Results

### VO_2max_ and Running Velocity

VO_2max_ was significantly lower in HYPO (43.6 ± 1.4 mL/kg/min) than in NOR (62.5 ± 1.2 mL/kg/min, *P* < 0.0001). The maximal running velocity during VO_2max_ test was significantly lower in HYPO (17.4 ± 0.1 km/h) than in NOR (19.6 ± 0.3 km/h, *P* < 0.0001). Consequently, the running velocity during each 3 min of the interval exercise (90% of VO_2max_) was significantly lower in HYPO (15.0 ± 0.2 km/h) than in NOR (16.4 ± 0.3 km/h, *P* < 0.0001).

### Metabolites, Blood Gas, and Plasma Volume Kinetics

**FIGURE 2:**
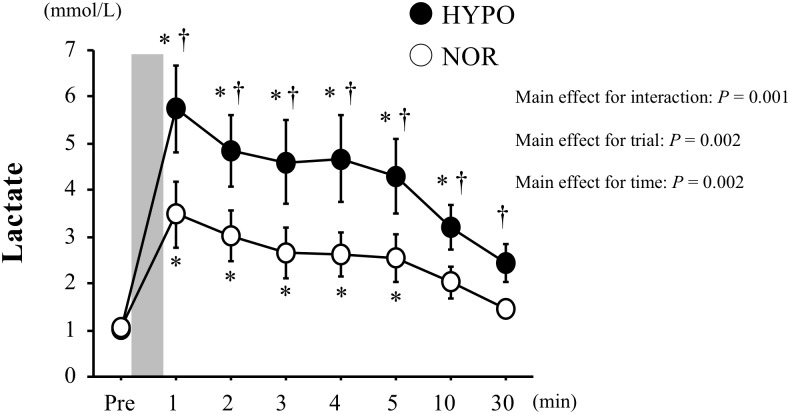
Changes in blood lactate concentrations before and after exercise. Values are means ± standard error (SE). The gray bar indicates the duration of exercise. ^∗^Significant difference compared with pre-exercise (Pre). ^†^Significant difference between NOR and HYPO.

The blood lactate concentration was significantly increased after exercise in both trials (main effect for time, *P* = 0.002). Moreover, it was significantly higher in HYPO than in NOR after exercise (interaction, *P* = 0.001; main effect for trial, *P* = 0.002, Figure 2).

The blood glucose concentrations increased significantly with exercise in both trials (main effect for time, *P* < 0.0001). However, there was no significant difference between the two trials at any time point. The serum insulin and ketone body concentrations did not change significantly over time in either trial. Moreover, no significant difference was observed between the two trials. In HYPO, the blood pO_2_ decreased significantly after exercise (main effect for time, *P* = 0.001), whereas the blood pO_2_ increased significantly after exercise in NOR (main effect for time, *P* = 0.001). Consequently, the blood pO_2_ remained significantly lower in HYPO after exercise compared with in NOR (interaction, *P* < 0.0001; main effect for trial, *P* < 0.0001). The blood pCO_2_ decreased significantly after exercise in both trials (main effect for time, *P* < 0.0001). The exercise-induced reduction in blood pCO_2_ was significantly greater in HYPO after exercise (interaction, *P* = 0.475; main effect for trial, *P* < 0.0001). The plasma volume decreased significantly after exercise in both trials. However, there was no significant difference between the two trials at any time point (Table 2).

### Acid–Base Balance

**FIGURE 3:**
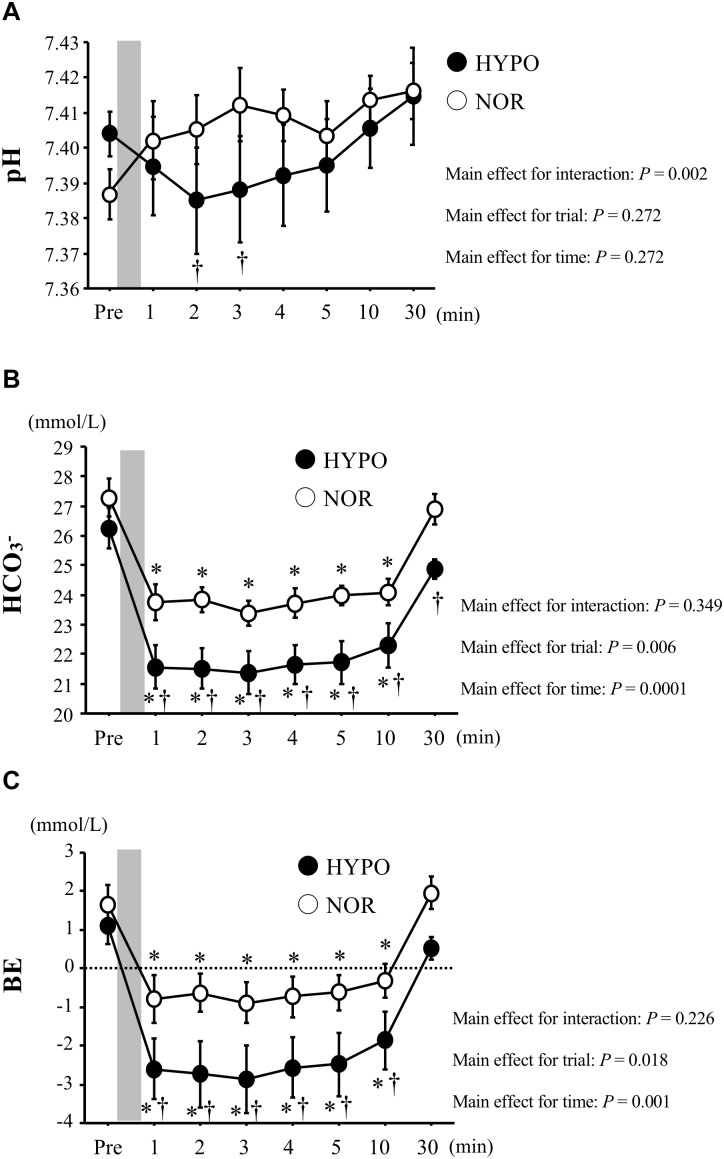
Changes in blood pH **(A)**, HCO_3_^−^**(B)**, and BE **(C)** concentrations before and after exercise. Values are means ± standard error (SE). The gray bar indicates the duration of exercise. ^∗^Significant difference compared with pre-exercise (Pre). ^†^Significant difference between NOR and HYPO.

After exercise, the blood pH was significantly lower in HYPO than in NOR (interaction, *P* = 0.002). The HCO_3_^−^ concentration decreased significantly after exercise in both trials (main effect for time, *P* < 0.0001). However, HYPO showed significantly lower HCO_3_^−^ concentrations after exercise (interaction, *P* = 0.434; main effect for trial, *P* = 0.002). The BE decreased significantly after exercise in both trials (main effect for time, *P* < 0.0001). The exercise-induced reduction in BE was significantly greater in HYPO after exercise (main effect for trial, *P* = 0.018, Figure 3).

### Blood K^+^

**FIGURE 4:**
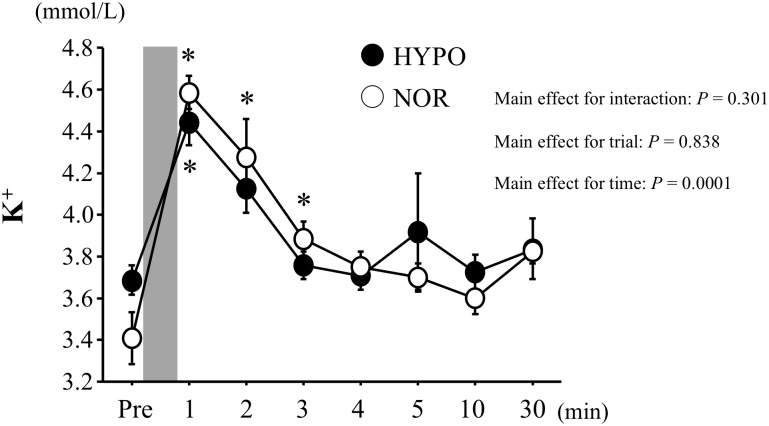
Changes in blood K^+^ concentrations before and after exercise. Values are means ± SE. The gray bar indicates the duration of exercise. ^∗^Significant difference compared with before exercise (Pre).

The blood K^+^ concentrations increased significantly after exercise in both trials (main effect for time, *P* < 0.0001). However, the exercise-induced elevations of blood K^+^ did not differ significantly between the two trials (Figure 4).

### Exogenous Glucose Oxidation Kinetics During Exercise

**FIGURE 5:**
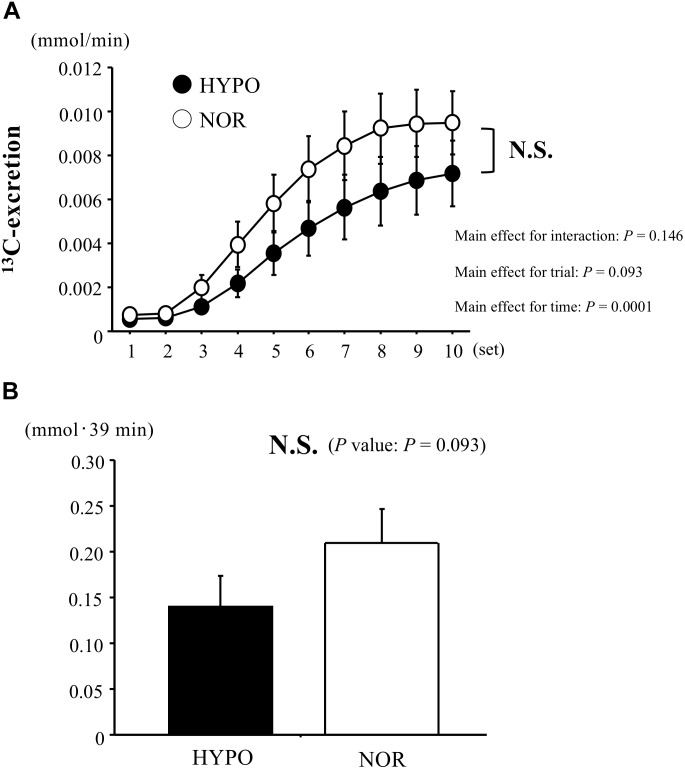
Changes in ^13^C-excretion **(A)** and area under the curve of ^13^C-excretion **(B)** during exercise. Values are means ± SE.

The ^13^C-excretion calculated by ^13^CO_2_/^12^CO_2_ increased during exercise in both trials (main effect for time, *P* < 0.0001), whereas there was no significant difference between HYPO and NOR (interaction, *P* = 0.146; main effect for trial, *P* = 0.09). The AUC for the ^13^C-excretion (during exercise) did not differ significantly between HYPO and NOR (*P* = 0.09, Figure 5).

### Cardiorespiratory Variables, Substrate Oxidation, and Energy Expenditure During Exercise

Table 1 shows the cardiorespiratory variables during exercise in each trial. VO_2_, VCO_2_, and SpO_2_ remained significantly lower in HYPO than in NOR throughout the exercise (main effect for trial, *P* < 0.0001). By contrast, HYPO showed a significantly higher RER during exercise compared with NOR (main effect for trial, *P* < 0.0001). During exercise, the VE and HR did not differ significantly between HYPO and NOR. The CHO oxidation during exercise was significantly higher in HYPO than in NOR (main effect for trial, *P* = 0.03). By contrast, HYPO showed a significantly lower fat oxidation (main effect for trial, *P* = 0.002) and EE (main effect for trial, *P* < 0.0001) during exercise compared with NOR.

### RPE

RPE-R and RPE-L did not differ significantly between HYPO and NOR during exercise.

## Discussion

As we hypothesized, the exercise-induced acidification of blood (i.e., lower blood pH and higher blood lactate) was significantly greater in HYPO than in NOR, although the running velocity was significantly lower in HYPO. By contrast, there was no significant difference in the exercise-induced elevation of blood K^+^ concentrations or ^13^C-excretion between HYPO and NOR.

**Table 1:** Cardiorespiratory variables, substrate oxidation and energy expenditure during exercise.

	NOR	HYPO	*p*-value
SpO_2_ (%)	95.0 ± 0.3	78.3 ± 1.1	*P* < 0.0001
VO_2_ (mL/kg/min)	52.1 ± 0.9	41.1 ± 1.6	*P* < 0.0001
VCO_2_ (mL/kg/min)	47.9 ± 1.2	42.1 ± 1.2	*P* = 0.0003
RER	0.92 ± 0.02	1.03 ± 0.02	*P* = 0.004
VE (L/min)	100 ± 6.0	105 ± 3.8	*P* = 0.315
HR (beats/min)	177 ± 2.4	176 ± 3.2	*P* = 0.545
CHO oxidation (g/min)	3.1 ± 0.3	3.7 ± 0.6	*P* = 0.044
Fat oxidation (g/min)	0.4 ± 0.1	0.1 ± 0.0	*P* = 0.002
EE (kcal/min)	15.7 ± 0.7	12.6 ± 0.5	*P* < 0.0001

*Values are mean ± SE.*

**Table 2:** Blood variables and plasma volume shift before exercise and during post-exercise.

		Pre	1	2	3	4	5	10	30 (min)
Glucose (mg/dL)	NOR	78 ± 1.5	94 ± 6.6^∗^	104 ± 6.1^∗^	103 ± 6.0^∗^	99 ± 4.3^∗^	100 ± 6.3^∗^	93 ± 6.0	78 ± 4.1
	HYPO	77 ± 2.2	101 ± 4.0^∗^	106 ± 4.7^∗^	102 ± 6.0^∗^	107 ± 5.0^∗^	101 ± 4.8^∗^	90 ± 4.8	77 ± 2.6
Insulin (μLU/mL)	NOR	2.64 ± 0.6	1.69 ± 0.4	–	–	–	–	–	3.40 ± 1.2
	HYPO	2.55 ± 0.6	1.93 ± 0.4	–	–	–	–	–	2.67 ± 0.6
Ketone body (μnol/L)	NOR	127.7 ± 48.0	96.2 ± 17.3	–	–	–	–	–	166.1 ± 58.7
	HYPO	88.7 ± 25.8	85.8 ± 6.2	–	–	–	–	–	76.7 ± 14.3
pO_2_ (mmHg)	NOR	49 ± 5.0	73 ± 3.2^∗^	85 ± 1.6^∗^	90 ± 1.8^∗^	90 ± 2.4^∗^	87 ± 3.2^∗^	77 ± 4.3^∗^	55 ± 5.5
	HYPO	61 ± 4.2	47 ± 1.4^†^	56 ± 1.3^†^	58 ± 1.1^†^	58 ± 1.4^†^	57 ± 1.7^†^	51 ± 2.0^†^	45 ± 3.6^†^
pCO_2_ (mmHg)	NOR	47 ± 1.4	39 ± 1.1^∗^	39 ± 0.5^∗^	38 ± 0.3^∗^	39 ± 0.5^∗^	40 ± 0.5^∗^	39 ± 0.7^∗^	43 ± 1.2
	HYPO	44 ± 1.7	36 ± 1.0^∗†^	36 ± 0.7^∗†^	36 ± 0.5^∗†^	36 ± 0.5^∗†^	36 ± 0.6^∗†^	36 ± 0.9^∗†^	40 ± 1.5
ΔPV (%)	NOR	0.0 ± 0.0	−12.7 ± 2.9^∗^	−12.5 ± 3.3^∗^	−7.0 ± 6.3^∗^	−11.3 ± 2.9^∗^	−9.8 ± 3.6^∗^	−4.3 ± 2.3^∗^	−3.4 ± 6.5
	HYPO	0.0 ± 0.0	−15.0 ± 2.9^∗^	−15.9 ± 2.7^∗^	−13.9 ± 2.2^∗^	−14.8 ± 2.3^∗^	−12.8 ± 2.4^∗^	−9.1 ± 2.0^∗^	−5.6 ± 2.2^∗^

*Values are means ± SE. ^∗^Significant difference vs. Pre. †Significant difference vs. Normoxia. PV, plasma volume.*

The exercise-induced blood lactate elevation was more profound in HYPO than in NOR. Moreover, HYPO caused significantly higher RER and CHO oxidation (evaluated by VO_2_ and VCO_2_) during exercise compared with NOR. These results indicate that carbohydrate metabolism during endurance exercise was augmented in hypoxia compared with in normoxia, which was consistent with previous findings (Katayama et al., 2010; Buchheit et al., 2012; Sumi et al., 2018a). The facilitated carbohydrate metabolism during endurance exercise under our HYPO condition may be explained by the increased ATP produced via the glycolytic system to compensate for the hypoxia-induced decline in ATP production via the aerobic system (Friedmann et al., 2007; Ogawa et al., 2007).

Many studies have evaluated carbohydrate metabolism during endurance exercise in hypoxia using traditional procedures, including blood glucose, lactate responses, and RER (Friedmann et al., 2004; Katayama et al., 2010; Morishima et al., 2014). Among these, the determination of RER during submaximal endurance exercise is most often used to monitor the substrate oxidation pattern, whereas carbohydrate oxidation may be overestimated by RER during endurance exercise in hypoxia due to the lower VO_2_ and hyperventilation (Ogawa et al., 2007; Ofner et al., 2014; Sharma et al., 2019). By contrast, we evaluated carbohydrate oxidation using a stable isotope (^13^C) as an alternative procedure to overcome this limitation. The consumed ^13^C-labeled glucose is oxidized mainly in working muscles during endurance exercise and is subsequently excreted in the expired gas as ^13^CO_2_. Therefore, the ^13^CO_2_/^12^CO_2_ ratio during endurance exercise reflects the amount of exogenous glucose oxidation (Harvey et al., 2007; Blondin et al., 2010; Smith et al., 2010; Tremblay et al., 2010). The ingestion of ^13^C-labeled glucose, specifically the exogenous glucose oxidation pattern in the tissues (e.g., skeletal muscle and liver), can be used to evaluate carbohydrate oxidation during endurance exercise in hypoxia. Moreover, the circulating blood during endurance exercise is predominantly distributed to working muscle; therefore, the augmented ^13^C-excretion during endurance exercise mainly reflects glucose oxidation in working muscle. Furthermore, we determined the serum ketone body concentrations (an indication of liver metabolism). However, there was no significant difference in the serum ketone body concentration over time, suggesting that energy metabolism in the liver (with a subsequent increase in serum ketone body concentration) was not augmented during the exercise. As previous studies revealed that endurance exercise in hypoxia promotes carbohydrate metabolism compared with exercise in normoxia (Katayama et al., 2010; Buchheit et al., 2012; Sumi et al., 2018a), we initially hypothesized that HYPO would elicit exogenous glucose oxidation during endurance exercise (i.e., increased ^13^C-excretion in HYPO compared with in NOR). However, no significant difference was observed in ^13^C-excretion between HYPO and NOR. Several factors are involved in exogenous glucose oxidation during exercise, but the EE during exercise strongly affects the amount of carbohydrate oxidation (Gautier et al., 1996). In our study, the EE during the 10 × 3 min run was significantly lower in HYPO than in NOR due to the lower running velocity. Therefore, future research should include comparisons involving the same EE in hypoxia and normoxia. Additionally, gastric emptying and the intestinal absorption of glucose also affect the exogenous glucose oxidation kinetics after oral consumption of labeled glucose. Unfortunately, the effects of exercise in hypoxia on gastric emptying and intestinal absorption remain unclear, although no study has reported that hypoxia altered gastric emptying or intestinal absorption.

Notably, the blood lactate elevation and CHO oxidation were significantly greater in HYPO, whereas ^13^C-excretion during endurance exercise did not differ significantly between the two trials. Because ^13^C-excretion during endurance exercise indicates exogenous glucose oxidation, the greater blood lactate elevation and CHO oxidation in HYPO may reflect facilitated muscle glycogen utilization (augmented endogenous glycogen utilization) during the exercise. The exercise in hypoxia promoted exercise-induced adrenaline elevation and sympathetic nerve activation (Wadley et al., 2006; Mazzeo, 2008; Katayama et al., 2010), and these promote muscle glycogenolysis during exercise (Watt et al., 2001; Wadley et al., 2006). Further research to address muscle glycogen utilization during endurance exercise in hypoxia would greatly aid our understanding of the physiological factors behind the augmented exercise-induced blood lactate elevation in hypoxia.

There was no significant difference in blood K^+^ levels between the two trials. Our initial hypothesis was that HYPO would augment the exercise-induced blood K^+^ elevation compared with NOR. Because lower pH increases the opening of K_ATP_ channels, it subsequently promotes K^+^ efflux from working muscle into the bloodstream (Davies, 1990; Nielsen et al., 2002). The accumulation of extracellular K^+^ has two possible explanations: (1) enhanced release of K^+^ from the working muscle and (2) decreased K^+^ re-uptake during exercise. During endurance exercise, the release of K^+^ commonly exceeds the K^+^ re-uptake, which consequently leads to the accumulation of K^+^ in the interstitium and blood. Unfortunately, the effects of exercise in hypoxia on the muscle Na^+^-K^+^-pump (K^+^ re-uptake) remain unclear. Street et al. (2005) determined the effects of sodium citrate ingestion on exercise-induced acidosis and potassium accumulation. The sodium citrate-ingestion group (CIT) had significantly higher blood and interstitial pH after exercise compared with a placebo water-ingestion group (PLA). Furthermore, CIT caused significantly lower exercise-induced interstitial K^+^ elevation compared with PLA (CIT: 8.0 mM, PLA: 11.0 mM). However, there was no significant difference in blood K^+^ concentrations between CIT and PLA (CIT, 4.1 mM; PLA, 4.2 mM). Therefore, due to the lack of data on the interstitial K^+^ response, caution is necessary when interpreting the K^+^ response in our study.

In our previous study (Sumi et al., 2018b), we investigated the effect of endurance exercise (cycling exercise) in hypoxia on acid-base balance and K^+^ responses. Consequently, endurance exercise in hypoxia caused higher blood pH and lower K^+^ concentrations compared with the same exercise in normoxia, which was not consistent with outcomes in the present study in spite of similar study design. However, the difference in absolute workload between hypoxia and normoxia was greater in the above previous study (approximately 20% lower in hypoxia) compared with the present study (approximately 10% lower in hypoxia). Furthermore, different exercise modality (cycling exercise in the previous study vs. running exercise in the present study) may explain different outcomes between the two studies.

In conclusion, high-intensity interval running in moderate hypoxia elicited decreased blood pH and elevated blood lactate despite the lower running velocity. However, it did not affect the exercise-induced blood K^+^ elevation or exogenous glucose oxidation. Therefore, our findings suggest that, despite the lower mechanical stress (lower running velocity), endurance exercise in moderate hypoxia causes higher metabolic stress and similar exercise-induced elevations of blood K^+^ and exogenous glucose oxidation compared with the same exercise in normoxia. The training in hypoxia may be an efficient training strategy to elicit training adaptations (e.g., improved CHO metabolism and muscle buffer capacity) with reduced risk of injury in lower legs.

## Author Contributions

DS and KG contributed to the study design, data collection, analysis, and manuscript writing. NK contributed to the data collection, analysis. HI contributed to the study design, data collection, and analysis. All authors read and approved the final manuscript.

## Conflict of Interest Statement

The authors declare that the research was conducted in the absence of any commercial or financial relationships that could be construed as a potential conflict of interest.
